# Impact of Data Presentation on Physician Performance Utilizing Artificial Intelligence-Based Computer-Aided Diagnosis and Decision Support Systems

**DOI:** 10.1007/s10278-018-0132-5

**Published:** 2018-10-15

**Authors:** L. Barinov, A. Jairaj, M. Becker, S Seymour, E. Lee, A. Schram, E. Lane, A. Goldszal, D. Quigley, L. Paster

**Affiliations:** 1 Koios Medical, New York, NY USA; 2 Princeton University, Princeton, NJ USA; 3 Rutgers University Robert Wood Johnson Medical School, New Brunswick, NJ USA; 4 University Radiology Group, East Brunswick, NJ USA

**Keywords:** Breast cancer, Machine learning, Artificial intelligence, Clinical workflow, Computer-aided diagnosis (CAD), Decision support

## Abstract

Ultrasound (US) is a valuable imaging modality used to detect primary breast malignancy. However, radiologists have a limited ability to distinguish between benign and malignant lesions on US, leading to false-positive and false-negative results, which limit the positive predictive value of lesions sent for biopsy (PPV3) and specificity. A recent study demonstrated that incorporating an AI-based decision support (DS) system into US image analysis could help improve US diagnostic performance. While the DS system is promising, its efficacy in terms of its impact also needs to be measured when integrated into existing clinical workflows. The current study evaluates workflow schemas for DS integration and its impact on diagnostic accuracy. The impact on two different reading methodologies, sequential and independent, was assessed. This study demonstrates significant accuracy differences between the two workflow schemas as measured by area under the receiver operating curve (AUC), as well as inter-operator variability differences as measured by Kendall’s tau-b. This evaluation has practical implications on the utilization of such technologies in diagnostic environments as compared to previous studies.

## Introduction

Excluding skin cancer, breast cancer has the highest incidence rate and the second highest mortality rate in women [1]. Early and accurate diagnosis is a cornerstone strategy used to minimize breast malignancy, morbidity, and mortality. Imaging plays a central role in diagnosis; specifically, digital mammography/tomosynthesis and ultrasound are the most frequently used screening and diagnostic modalities. In current imaging protocols, ultrasound (US) is a valuable tool for evaluating breast tissue, achieving sensitivity comparable to digital mammography (DM) and improved detection of invasive and node-negative breast cancers [2]. This improvement, however, comes at the cost of lower PPV3 and specificity [3]. In practice, the increased false-positive rate manifests as an increase in benign biopsies.

One avenue being explored to address these concerns is the introduction of machine learning-based artificial intelligence (AI) systems. While these systems have been utilized in the past for mammography, their benefits have been recently called into question [4]. Additionally, rather than aiding in diagnosis, these systems have traditionally been used as an aid for the detection of suspicious areas [5–7]. This approach has been replicated in automated whole breast ultrasound (ABUS) but is only cleared to target areas not known to have suspicious findings [8].

More recently, tools have been developed to aid the diagnostic performance of radiologists, offering automated assessments of lesion characteristics and risk. Initial iterations have demonstrated a meaningful increase in sensitivity but a large decrease in specificity [4]. As machine learning techniques have progressed over the last 6 years, however, advances in performance within the diagnostic ultrasound space have followed suit.

A recent study demonstrated that integration of US with a new AI-based decision support (DS) system offers substantial improvement in both sensitivity and specificity. When tested alone, the DS platform was shown to exceed radiologist performance in US data analysis, showing a 34–55% potential reduction in benign biopsies and an increase in the positive predictive value of lesions sent for biopsy (PPV3) of 7–20% [9].

While the system’s raw performance is promising, DS’s practical efficacy and impact also need to be assessed when integrated into existing real-world clinical workflows. This study investigates the clinical impact of two different diagnostic workflows. Clinical impact is evaluated as a function of how diagnostic support is presented. Presentation can either be sequential, where the clinician has an initial opportunity to evaluate the case unaided before receiving DS, or independent, where the case and decision support are presented together. Stand-alone clinician accuracy is compared to that of a clinician utilizing DS, and the system’s impact on intra-operator and inter-operator variability is evaluated. The goal of this study is to evaluate workflow schemas for DS integration and their effects on diagnostic accuracy.

## Methods

### Data Collection

**Fig. 1:**
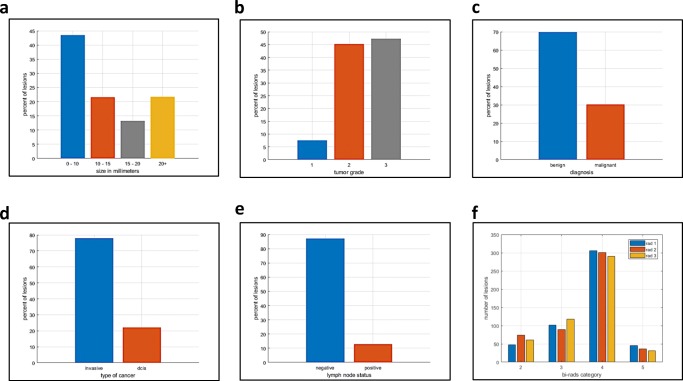
Lesion population statistics. **a** Tumor size. **b** Tumor grade. **c** Benign or malignant. **d** DCIS (non-invasive) vs invasive. **e** Lymph node status. **f** BI-RADS designation for the three radiologists tested

**Fig. 2:**
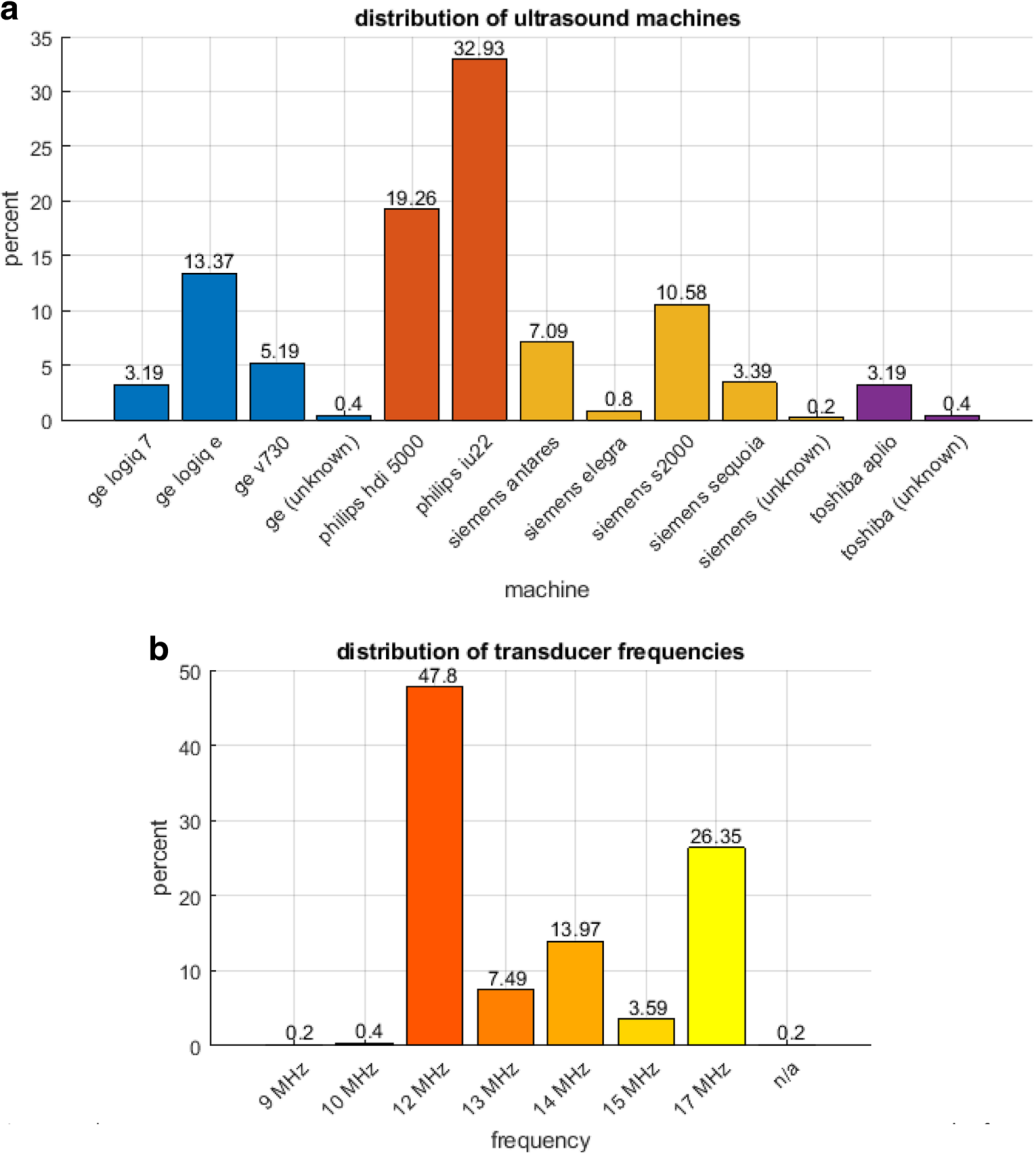
Ultrasound equipment characteristics. **a** Manufacturer. **b** Transducer frequency. “n/a” refers to cases in which US transducer frequency was not recorded

Using data acquired from the ACRIN 6666 trial [10], 500 cases were identified for inclusion. Lesion population statistics can be seen in Fig. 1. The dataset was enriched for malignancy, while all other statistics were chosen to approximate current population numbers per the Breast Cancer Research Foundation (BCRF) [10]. All pathological ground truth for malignant lesions came from biopsy-proven pathological follow-up, while for benign lesions, ground truth was established via biopsy or 1 year follow-up if the lesions were BI-RADS 4 and above or BI-RADS 3 and below, respectively. This dataset includes images obtained using a diverse set of US equipment and a range of high-frequency breast transducers (Fig. 2). Overall, this equipment and the lesion evaluated in the dataset accurately represent current clinical practice including inclusion of cases from both academic and non-academic sites as well as dedicated breast and non-dedicated imaging centers [10].

### Machine Learning

Utilizing original radiologist-designated regions of interest (ROI), two orthogonal views of each lesion were used to generate a machine learning system-based score [9]. These ROIs were selected by the radiologist who had originally read the case clinically. The score ranges from 0.0 to 1.0 and is subdivided into four categories, each representing a clinical course of action (Table 1).

**Table 1:** Score ranges and their corresponding categorical outputs. These ranges and categories are inherent to the system and were not designed or altered for this study

Categorical output	Score range
Benign	[0, 0.25)
Probably benign	[0.25, 0.5)
Suspicious	[0.5, 0.75)
Malignant	[0.75 1.0]

**Fig. 3:**
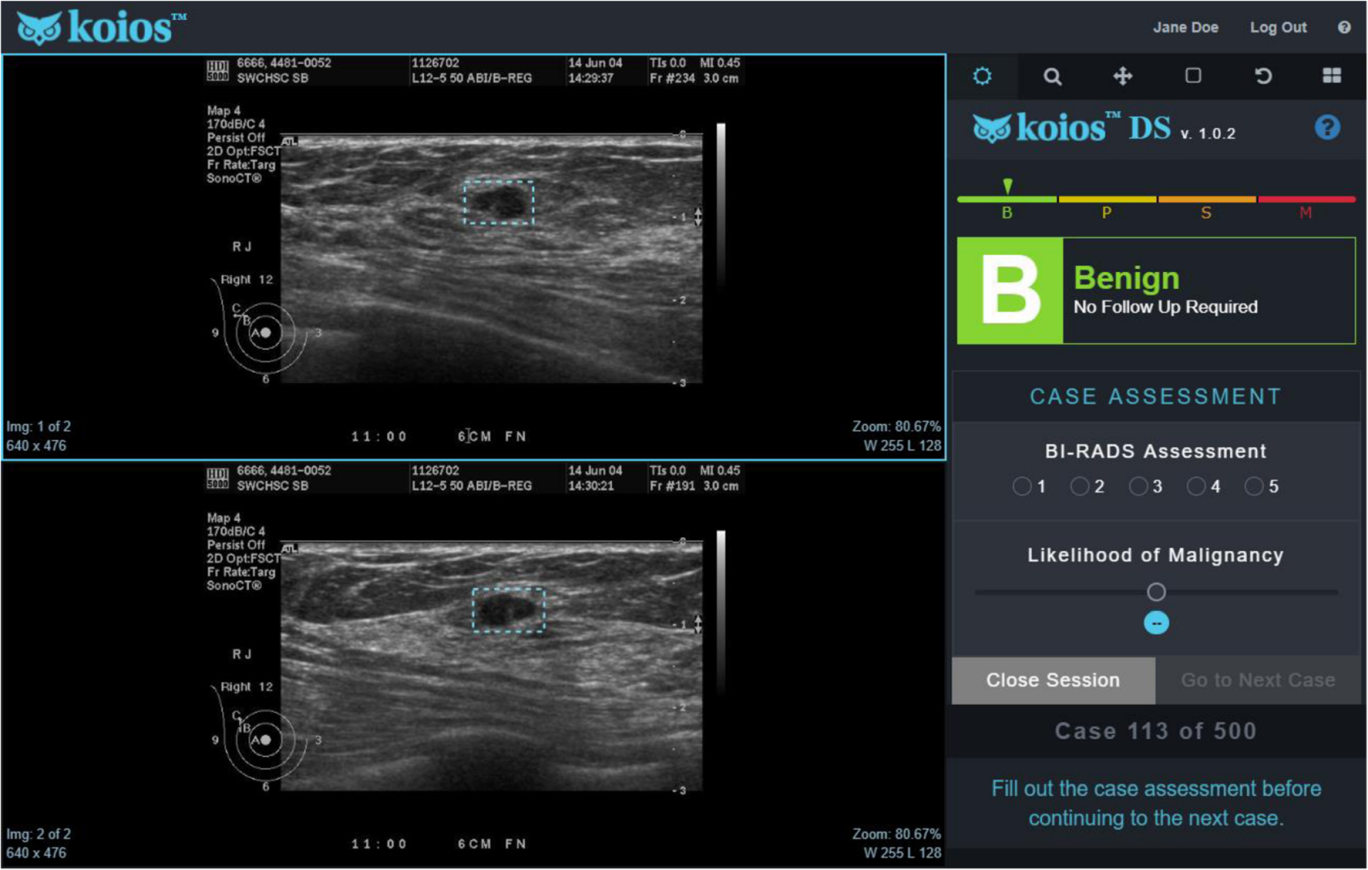
Screen capture of the study platform. The left side shows two orthogonal views with ROIs. On the right side is the DS output and the radiologist case assessment input (BI-RADS assessment and likelihood of malignancy percentage)

These scores were presented to all study readers in a graphical form in the electronic Case Report Form (eCRF) (Fig. 3).

### Reader Characteristics and Training

Three American Board of Radiology (ABR) certified radiologists, training and experience summarized in Table 2, evaluated the 500 case dataset. Each radiologist was initially given a 30-min training session to understand the machine learning output, stand-alone performance of the system, and the eCRF form. After training, each radiologist demonstrated proper utilization of the study platform by assessing 10 test cases proctored by the training staff.

**Table 2:** This table provides a summary of the three readers involved in this study

Radiologist ID	Post-educational training experience (years)	ABR certified	Breast fellowship training
1	20+	x	x
2	10+	x	x
3	5+	x	

Each reader specializes in breast imaging and performs a mix of screening and diagnostic breast imaging across multiple modalities.

### Reader Workflow

Cases were presented to and scored by radiologists in a single software environment that showed orthogonal images, ROIs, and the DS output. The study platform then queried the radiologist to input a Breast Imaging Reporting and Data System (BI-RADS) score (the current clinical system used to evaluate lesion suspicion) and likelihood of malignancy (LoM) as a percentage (Fig. 3).

**Fig. 4:**
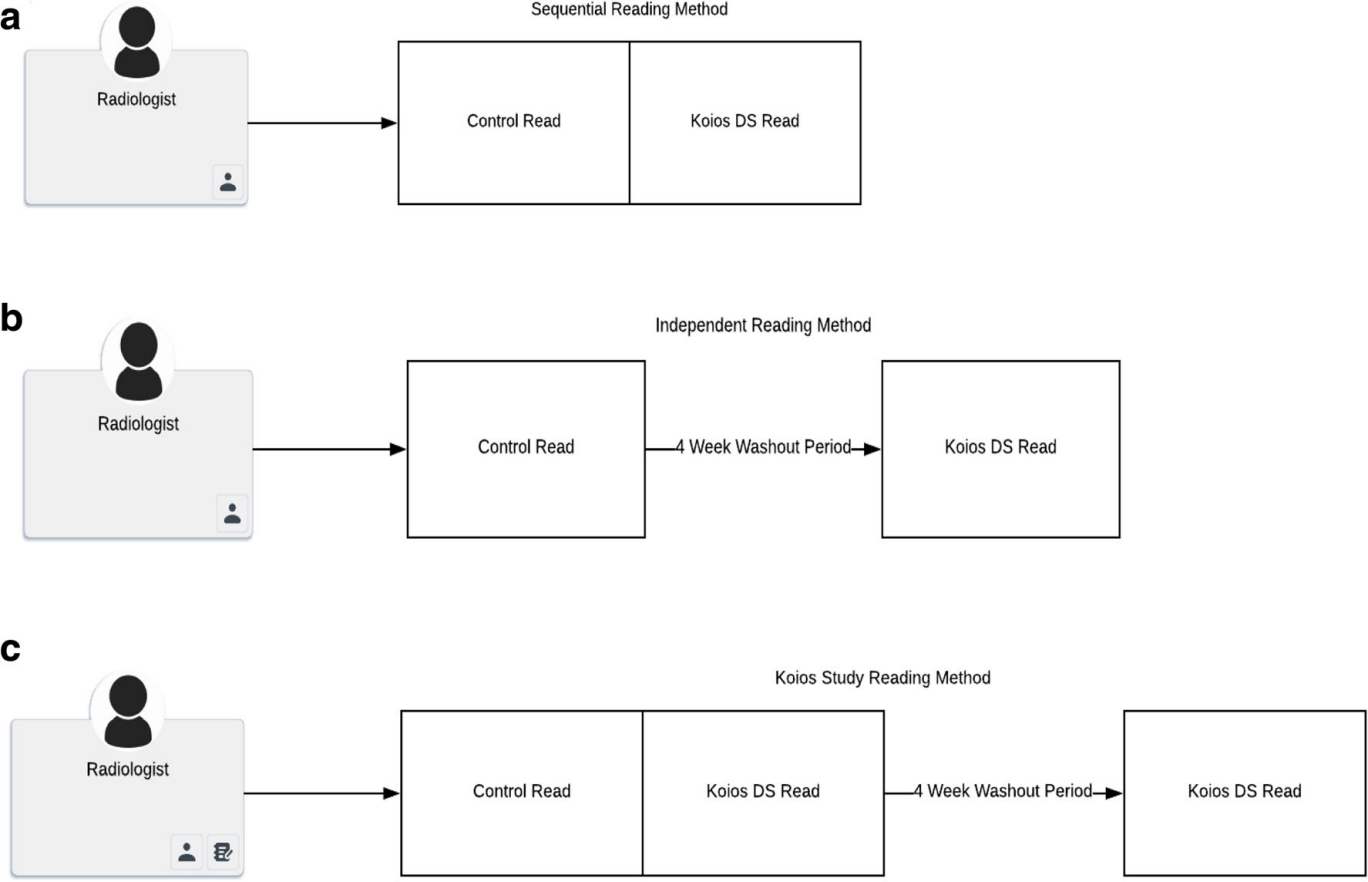
Schematic representation of the **a** sequential and **b** independent reading paradigms. A combination approach seen in **c** was utilized in this study

Using this software, the readers reviewed ultrasound images using two separate workflows which are summarized in Fig. 4:

### Sequential workflow

1. Readers reviewed the case without the DS output (control read)
2. Readers scored the case via BI-RADS and LoM
3. DS output was presented to the readers
4. Readers re-scored the case via BI-RADS and LoM

### Independent Workflow

This workflow was employed after a 4-week “washout” period that followed the sequential workflow. After this washout period, readers where shown the images and the DS output, and they scored the case. In the independent workflow, readers were presented with 450 cases in randomized order. Fifty cases were withheld and reserved as control cases in order to measure intra-reader variability after the 4-week washout period. This workflow is summarized in the following sequence:

Readers were presented with one of the following:

1. Control case read with no DS output (50 Total)
  - Readers scored the case via BI-RADS and LoM
**OR**
2. A case containing DS output (450 Total)
  (b) Readers scored the case via BI-RADS and LoM

### System Evaluation

Radiologists were presented with DS output that used the original ROIs selected during clinical evaluation of the cases. This choice was made to enhance reader efficiency so that a high volume of cases could be evaluated in a practical time frame. However, while it is felt that the ROI a radiologist chooses manually would be very similar to the ROI used in this study, it is possible that results would be impacted the variation caused by manually demarcated ROIs. To evaluate the system’s robustness to variation in the ROI boundaries, two assessments were performed.

First, DS output’s robustness to ROI boundary variation was assessed by evaluating the 500 cases 20 times, randomly varying the ROI boundary each time. Specifically, each corner of the region was shifted at random by up to 20% in each dimension from the predetermined optimal cropping. ROC curves and the AUC distributions were calculated.

Second, the boundaries between BI-RADS 3 Probably Benign (P) and BI-RADS 4a Suspicious (S) represents the most clinically impactful diagnostic decision point. It is critical to understand the effects of ROI variability on class switching across categories, and specifically the P-S boundary. Since the system’s output is categorical, changes across this decision boundary have the potential to change clinical management. The level of class switching due to ROI sensitivity was evaluated by utilizing the 20 ROIs generated in the previous analysis and counting the number of times category switching is observed compared to the initial radiologist supplied cropping.

Finally, in order to verify the relationship between the system’s categorical output and BI-RADS, a sensitivity and specificity analysis was conducted. For each categorical group, the sensitivity and specificity values were compared between the system and each participating reader’s BI-RADS assessment.

### Reader Evaluation

The area under the receiver operating curve (AUC) for each radiologist across each reading paradigm was calculated and compared to their control reads. The estimate of the change in AUC as well as the 95% confidence intervals was made by using the Dorfman-Berbaum-Metz method of MRMC analysis using the Metz-ROC LABMRMC software [11].

Intra- and inter-operator variability was assessed via Kendall’s tau-b correlation coefficient [12]. This assessment was done in a pairwise fashion across each pair of readers before and after being provided with the decision support output and across each reading methodology.

## Results

**Fig. 5:**
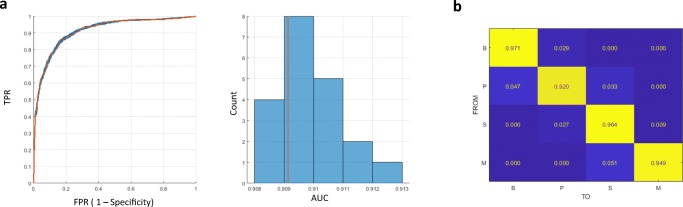
Results of the system evaluation. **a** ROC curves and corresponding AUCS assessing impact of ROI boundary variation. **b** Assessment of class switching due to ROI boundary variation

**Fig. 6:**
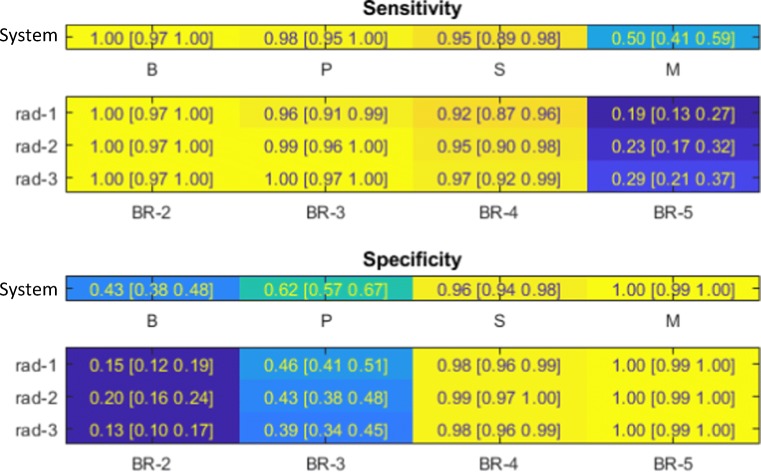
Sensitivity and specificity of each reader’s BI-RADS grading is compared to that of the systems corresponding categorical output

System Evaluation: Variability in ROI boundary produces no significant change in either the shape of the ROC curve or the AUC values (Fig. 5a). Similarly, the ROI analysis shows minimal class switching between P-S/S-P categories, 2.7 and 3.3%, respectively (Fig. 5b). We further correlate the results for each of the categorical groups supplied by the system to the BI-RADS assessments provided by the radiologist readers (Fig. 6).

Analysis of the operating points of the system appear to be as good, or better, than the radiologists tested in this study (Fig. 6). This suggests that the performance of categorical outputs of the system align to and exceed the performance of the BI-RADS assessments.

The reader evaluation analysis showed a system only AUC [95% confidence interval (CI)] of 0.8648 [0.8345–0.8893]. Each radiologist’s stand-alone performance is detailed in Table 3.

**Table 3:** Each reader’s performance was assessed prior to being presented the system’s output. The results of their control reads as measured via AUC is shown in this table

Radiologist ID	AUC, 95% CI
1	0.7618 [0.7244–0.7934]
2	0.7543 [0.7197–0.7887]
3	0.7325 [0.6897–0.7689]

**Fig. 7:**
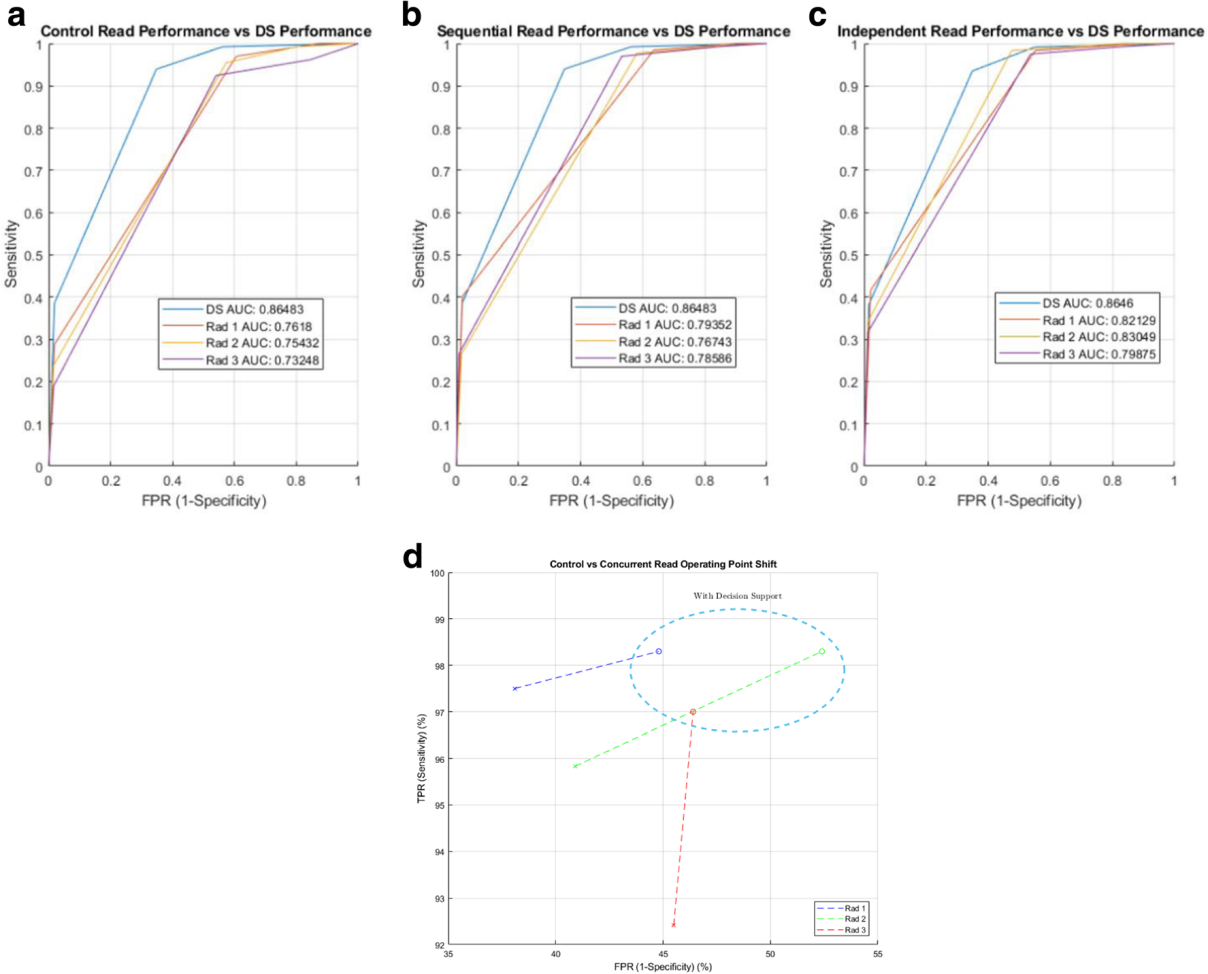
Comparative assessment of **a** control, **b** sequential, and **c** independent reading workflows. Operating point specific improvement for Independent vs control assessments were additionally measured (D)

Similarly, the sequential and independent joint performance is summarized in Table 4 and Fig. 7.

**Table 4:** In order to compare the two reading methodologies, the readers’ performance was assessed via AUC compared to their control reads summarized in Table 3. None of the readers attained statistical significance when utilizing sequential reads, while all readers were significantly better when utilizing an independent reader strategy

Radiologist ID	Sequential read AUC, 95% CI	*P* value CR vs SR two-tailed alpha = 0.05	Independent read AUC, 95% CI	*P* value CR vs IR two-tailed alpha = 0.05
1	0.7935 [0.7567–0.8229]	0.235	0.8213 [0.7861–0.8516]	0.0285*
2	0.7674 [0.7327–0.8001]	0.601	0.8305 [0.7982–0.8594]	0.00155*
3	0.7859 [0.7527–0.8174]	0.0532	0.7988 [0.7632–0.8310]	0.0160*

*Significant

Intra-reader and inter-reader variability, as measured by Kendall’s tau-b, is summarized in Tables 5 and 6, respectively.

**Table 5:** To further characterize reader performance, intra-reader variability was measured via Kendall’s tau-b

Radiologist ID	Kendall’s tau-b for intra-reader variability assessment.
1	0.597
2	0.595
3	0.529

**Table 6:** Pairwise combinations of variability were measured utilizing the sequential read (SR) methodology. Interestingly, the independent read (IR) variability was lower than intra-reader variability

Radiologist ID (KTB for reading methods)	1 (control, SR, IR)	2 (control, SR,IR)	3 (control, SR,IR)
1 (control, SR,IR)	(1, 1, 1)	(0.5505, 0.6263*, 0.7231**)	(0.4944, 0.6640*, 0.7476**)
2 (control, SR,IR)	–	(1, 1, 1)	(0.4229, 0.5641*, 0.6231**)
3 (control, SR,IR)	–	–	(1, 1, 1)

*Significant with *p* < .01; **significant difference with *p* < 1e−8

## Discussion

The current study confirms that this DS system performs favorably when compared with a radiologist’s performance, confirming prior studies [9]. In addition, rather than simply comparing DS system performance to that of a radiologist, it extends these prior results by assessing workflows that more realistically approximate clinical practice. Past studies have shown mixed evidence on the effect of the tested reading methodologies on overall reader performance, but none have conducted an investigation within the context of diagnostic decision support, and their effects remain unknown [13]. Our results show a sizeable variation in performance obtained depending on which reading methodology was chosen (Table 4). The impact of the reading methodology on study performance has practical workflow considerations. These results suggest there may a strong impact of confirmation bias in a sequential study design. This can be clearly seen in Fig. 7b versus Fig. 7c, where the deviation from the control assessment is significantly smaller in the sequential read versus independent read. This has practical implications in the utilization of machine learning decision support in breast ultrasound diagnostics and likely beyond.

Furthermore, as would be expected by providing a supplemental, concurrent read that out-performs the original reader, overall inter-operator variability decreased significantly. Surprisingly, inter-operator variability decreased even beyond that of the 4-week washout intra-reader variability per Tables 5 and 6. Due to the study design, the effects of a machine learning-based concurrent read on intra-operator variability were not evaluated, but with the evidence presented in this study, it would seem likely that a proportional decrease in this metric could be expected.

### Clinical Applications

When looking at practical clinical workflow applications, the performance results and study design have a number of implications on the application of AI software.

In clinical practice, the typical sequence of radiology workflow is:

1. Radiologist assesses mammographic images and instructs technologist to perform a complete mammographic examination
2. Radiologist decides if US is needed
3. If so, the technologist acquires images and presents to radiologist
4. Radiologist assesses images and the radiologist may or may not confirm the results with radiologist real time scanning
5. Radiologist formulates diagnosis

In the sequential workflow, the radiologist would complete “step 5” then assess the DS output. In the independent workflow, the DS output would be presented to the radiologist during “step 4,” along with the US images (e.g., along with the other US “data”).

In clinical practice, if the radiologist has confidence in the DS system, the independent workflow seems more likely to impact clinical management, e.g., the radiologist looks at all the data (demographic/clinical history, mammographic, US, DS output) and forms a diagnosis (the ultimate goal).

### Comparison to Other Systems

Prior research has explored the difference between sequential and independent study designs and their respective effects on performance [13–16]. These studies have suggested that both designs produce similar performance results within comparative analyses. They then conclude that sequential designs are preferable due to lower time and resource requirements. Interestingly, our results seem to suggest a more significant deviation between these two study designs.

The difference between our results and the results discussed above can perhaps be attributed to the following factors. The first consideration which must be made is that the technology being tested and the underlying modality are both different. Second, the task being performed by the decision support system is all-together different from the CAD systems being examined in these studies. Most of the systems under study are focused on detection, while DS is focused on diagnosis.

In detection, a sequential read implies that the clinician identifies regions of interest, performs CAD, and then potentially examines additional regions, as suggested by the CAD device. When performing an independent read, that clinician will see regions of interest as identified by CAD and may then examine additional regions based upon their own inspection. In both cases, the clinician is combining their detection results with the CAD’s, so it is reasonable that performance is similar between the two.

In diagnosis, a sequential read implies that the clinician will examine a nodule, arrive at a conclusion, and then receive the recommendation of the DS system. The recommendation will either confirm their decision, alter their decision, or not be considered. In an independent read, the recommendation is presented at the outset, and is considered as additional input data alongside the image as the clinician makes a decision. In the sequential case, since the clinician has already formed an opinion, a dissenting recommendation may meet with intransigence, and wind up suffering from confirmation bias.

### Limitations

While the results suggest a strong performance benefit, there are several limitations to the study design that must be taken into consideration. The study only consisted of three (3) readers. Although the readers had varying degrees of experience within the study, the study does not capture the full breadth of readers across the broader population of radiologists that read and interpret breast ultrasound images. The number of cases (500) and enrichment within the study may also limit its ability to represent the typical distribution of cases that a reader would expect to see in practice. The choice of distribution of the cases was an attempt to create a set that was broadly representative of the greater populations of lesions as a whole but could also answer questions in a statistically meaningful way about events that have low population incidence. This further extends to the retrospective nature of the study design. In clinical practice, additional factors impact clinical reasoning that are not fully represented in the study, such as patient history, prior studies, and patient preference towards clinical follow up. While a prospective, randomized control study would have addressed some of these concerns, it would come at the cost of study time and complexity.

This study did not compare intra-operator variability with and without a decision support output as it would have required an additional set of reads for each participant. Based on the current results, it would seem likely that the intra-operator variability would decrease, but without study modification, the occurrence and extent of the variability is unknown.

The first reading session of this study was not counterbalanced, and all readers initially read sequential first and independent second. Cases without a corresponding DS output were randomly introduced in the independent session to break up the reading schedule and allow for the evaluation of intra-operator variability. The lack of counterbalancing between sequential and independent reads may have introduced slight reader bias when comparing the two paradigms.

Finally, the current study assesses the impact of DS on US interpretation in isolation, when in fact a complete breast imaging assessment incorporates demographic data, clinical history, and other imaging modalities such as mammography. New and exciting avenues of inquiry are needed to more fully evaluate the role and utility of DS needs in this larger context.

## Conclusion

We have been able to demonstrate that reader workflow can significantly affect clinical performance when incorporating AI-based decision support tools. This evaluation has novel practical implications on the utilization of such technologies in diagnostic environments as compared to previous studies which have concluded an effective equivalence between these two reading paradigms. Independent reads (concurrent reads) have shown dramatic shifts in reader performance and inter-operator variability as compared to either control reads or sequential reads. The evidence provided in this study can be used to impact both study design when demonstrating efficacy of new diagnostic decision support tools, as well as their implementation in practical environments.
